# dVGLUT Is a Mediator of Sex Differences in Dopamine Neuron Mitochondrial Function Across Aging and in a Parkinson's Disease Model

**DOI:** 10.1111/acel.70096

**Published:** 2025-05-12

**Authors:** Silas A. Buck, Samuel J. Mabry, Tenzin Kunkhyen, Zilu Yang, Sophie A. Rubin, Jinting Yang, Claire E. J. Cheetham, Zachary Freyberg

**Affiliations:** ^1^ Center for Neuroscience University of Pittsburgh Pittsburgh Pennsylvania USA; ^2^ Department of Psychiatry University of Pittsburgh Pittsburgh Pennsylvania USA; ^3^ Department of Neurobiology University of Pittsburgh Pittsburgh Pennsylvania USA; ^4^ Department of Cell Biology University of Pittsburgh Pittsburgh Pennsylvania USA

**Keywords:** aging, dopamine, *Drosophila*, dVGLUT, glutamate, mitochondria, Parkinson's disease, reactive oxygen species

## Abstract

Sex differences in Parkinson's disease (PD) offer insights into mechanisms of dopaminergic cell resilience. Female dopamine (DA) neurons are more resilient via mechanisms that remain unclear. Here, we discovered key sex and regional differences in mitochondrial generation of cytotoxic reactive oxygen species (ROS) and their implications for DA neuron resilience using the *Drosophila* model. While aging raised mitochondrial ROS in DA neurons of both sexes, we observed a sexually dimorphic response in the paraquat (PQ) PD model. DA neuron knockdown of the *Drosophila* vesicular glutamate transporter (dVGLUT) increased mitochondrial ROS only in males, leaving females protected. Cell depolarization, a physiological stressor, similarly raised mitochondrial ROS in DA neurons selectively in males following dVGLUT knockdown. We also identified dVGLUT‐dependent changes in intracellular ATP in both sexes. Overall, we discovered sexually dimorphic relationships between dVGLUT, ATP synthesis, and ROS generation in DA neurons, providing a mechanistic basis for DA neuron resilience.

## Graphical abstract

We examined sex and regional differences in mitochondrial reactive oxygen species (ROS) generation and their implications for dopamine (DA) neuron resilience using the *Drosophila* model. We found: (1) higher basal ROS in males versus females; (2) age‐related ROS increases in both sexes; and (3) DA neuron‐specific dVGLUT RNAi knockdown raised ROS in males after either paraquat treatment or cell depolarization, while females showed no changes.

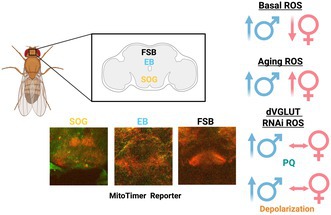

## 1 Introduction

Mitochondrial energy generation is fundamental for fueling the metabolic needs of all eukaryotic cells, including neurons. However, during ATP synthesis, the flux of electrons down the electron transport chain concomitantly generates cytotoxic reactive oxygen species (ROS) as an unavoidable byproduct (Kowalczyk et al. 2021; Shadel and Horvath 2015; Zhao et al. 2019; Adam‐Vizi 2005; Cadenas 2018). Indeed, while ROS can accumulate from several sources including NADPH oxidases (Lambeth 2004), an estimated 90% of ROS are generated by mitochondria (Balaban et al. 2005). This buildup of mitochondrial ROS has been implicated in cellular dysfunction during aging (Balaban et al. 2005; Giorgi et al. 2018). Furthermore, increased levels of oxidative stress and ROS are common factors in multiple aging‐related neurodegenerative diseases, including Parkinson's disease (PD) (Kim et al. 2015; Singh et al. 2019). This is especially relevant since PD is one of the most prevalent neurodegenerative diseases today (Dorsey et al. 2018) and is characterized by selective dopamine (DA) neuron loss. Notably, not all midbrain DA neurons are equally affected by PD: DA neurons in the substantia nigra *pars* compacta (SNc) are particularly vulnerable while the DA neurons in the ventral tegmental area (VTA) are more protected (Sulzer and Surmeier 2013; Surmeier et al. 2017; Hirsch et al. 1988; Damier et al. 1999; Steinkellner et al. 2018; Alberico et al. 2015). The mechanisms for these region‐specific differences in vulnerability are unclear and remain an area of active study.

In mammals, the vesicular glutamate transporter 2 (VGLUT2) has been identified as a marker for cells that are selectively resilient to neurodegeneration in several PD models (Dal Bo et al. 2008; Steinkellner et al. 2022; Shen et al. 2018). This unique population of dopaminergic cells that co‐express VGLUT2 is more likely to survive in preclinical models of PD compared to DA neurons without VGLUT2 (Steinkellner et al. 2018; Shen et al. 2018). Notably, the VTA has a higher proportion of VGLUT2‐expressing DA neurons when compared to the SNc, offering a potential explanation for the VTA's greater resilience in PD (Poulin et al. 2018; Yamaguchi et al. 2015; Maingay et al. 2006). Consistent with this, a recent postmortem human brain study demonstrated that VGLUT2‐expressing midbrain DA neurons are more resilient in PD patients (Steinkellner et al. 2022), indicating this mechanism's clinical relevance. These findings are also evolutionarily conserved since *Drosophila* similarly possess a subpopulation of DA neurons that co‐express a VGLUT2 ortholog, dVGLUT (Buck et al. 2021a; Aguilar et al. 2017). As in mammals, reducing dVGLUT expression in DA neurons eliminates the greater resilience of these neurons in adult female flies throughout the aging process (Buck et al. 2021a). Clues concerning the mechanisms by which dVGLUT/VGLUT2 modulates DA neuron resilience come from our earlier work, demonstrating that a key physiological role for both dVGLUT and VGLUT2 is to modulate activity‐dependent DA vesicle loading to tune vesicular content during periods of cell firing (Aguilar et al. 2017). Nevertheless, the mechanisms underlying the protection conferred to DA neurons by VGLUT2 or dVGLUT are still unknown.

Important mechanistic clues come from comparative studies showing sex differences in DA neuron VGLUT2/dVGLUT expression where females express more VGLUT2 (or dVGLUT) in DA neurons compared to males in rodents, flies, and humans (Buck et al. 2021a). Despite most work on PD rodent models having been performed in males (De Miranda et al. 2019), it has been demonstrated recently that females are more resilient to DA neurodegeneration in models of PD (De Miranda et al. 2019; Dluzen et al. 1996) and aging (Buck et al. 2021a). Just as importantly, clinical PD is more common in men than in women (Ben‐Shlomo et al. 2024). Such sex differences in DA neuron resilience can provide novel insights for uncovering mechanisms of DA neuroprotection. Together, these data point to the possibility that dVGLUT/VGLUT2 is one of the main drivers behind DA neuron resilience both in aging (Buck et al. 2021a) and in PD (Steinkellner et al. 2018, 2022; Shen et al. 2018) and could contribute to the observed regional and sex differences in DA neurodegeneration.

In this study, we utilized the genetically tractable *Drosophila* model to elucidate the mechanisms for dVGLUT/VGLUT2‐driven DA neuron resilience. We demonstrate sex differences in basal mitochondrial ROS levels, with females having lower ROS in dopaminergic projections throughout several key adult fly brain regions. Furthermore, we show that aging increases DA neuron mitochondrial ROS in both males and females across brain regions. Interestingly, we discovered that dVGLUT is a key modulator of mitochondrial ROS in the paraquat (PQ) model of PD in *Drosophila*. Critically, PQ exposure causes DA neurodegeneration by generating ROS that damage mitochondrial function (McCormack et al. 2002; Castello et al. 2007; Aryal and Lee 2019). We also demonstrate that dVGLUT is a physiological modulator of activity‐driven changes in intracellular ATP levels as well as mitochondrial ROS in response to neuronal depolarization. Overall, our data implicate DA neuron dVGLUT as a key modulator of ATP production‐related mitochondrial ROS in response to physiological and pathological cellular stress.

## 2 Results

### 2.1 MitoTimer Is a Biosensor of Mitochondrial Reactive Oxygen Species in Dopamine Neurons

**FIGURE 1:**
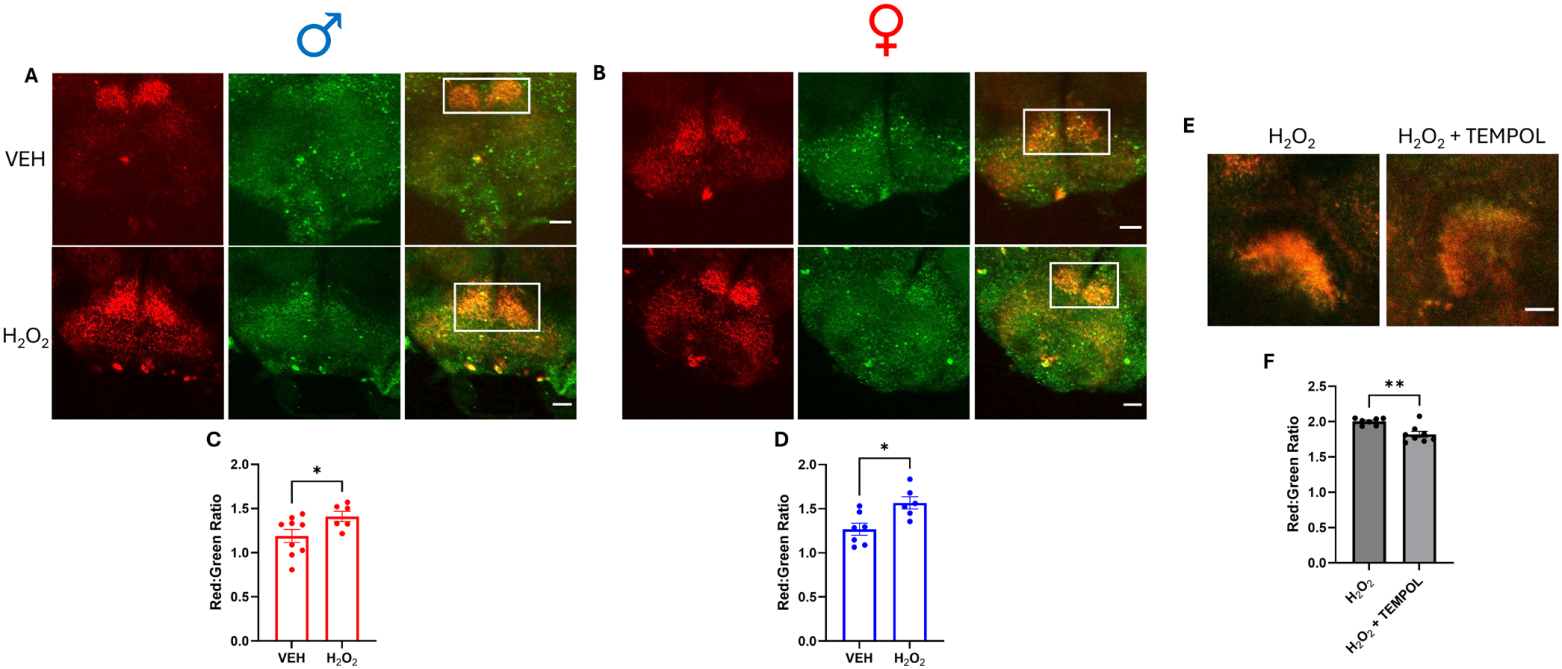
H_2_O_2_ treatment increases mitochondrial reactive oxygen species (ROS) in both males and females. (A, B) Representative images of dopamine (DA) neuron projections to the suboesphageal ganglion (SOG) region labeled by the ratiometric MitoTimer ROS biosensor in adult *Drosophila* brains of male (A) and female (B) control flies; images represent the overlap between MitoTimer red and green fluorescence in the SOG (white box). Flies were exposed to either H_2_O_2_ (3%, 2d) or vehicle (VEH). Scale bars = 25 μm. (C and D) Quantification of MitoTimer red:green ratios within the dopaminergic projections to the SOG revealed a significant increase in signal in response to H_2_O_2_ compared to VEH in both male (C; *t* = 3.053, *p* = 0.011, *n* = 6–7) and female (D; *t* = 2.186, *p* = 0.048, *n* = 6–9) flies. (E) Representative images of MitoTimer‐labeled dopaminergic projections to the fan‐shaped body (FSB) in male control flies. Flies were exposed to either TEMPOL (3 mM, 5d) or TEMPOL in conjunction with H_2_O_2_ (3%, 2d). (F) Quantification of MitoTimer red:green ratios in DA neuron projections to the FSB revealed a significant reduction of signal in the H_2_O_2_ + TEMPOL group compared to H_2_O_2_ alone (*t* = 3.670, *p* = 0.002, *n* = 7–8). Data are represented as means ± SEM. Student's unpaired *t*‐test (C, D, F). *p < 0.05; **p < 0.01.

MitoTimer, a mitochondria‐targeted fluorescent ROS biosensor, is composed of a mitochondrial matrix‐localized dsRed mutant that shifts its emission from green to red fluorescence upon local ROS‐mediated oxidation (Laker et al. 2014). This fluorescence shift, represented as the red:green ratio, enables MitoTimer to function as a reporter of either acute or steady‐state levels of mitochondrial ROS, in addition to its established role in measuring mitochondrial turnover (Hernandez et al. 2013; Hu et al. 2023). We specifically expressed MitoTimer in DA neurons using the promoter of tyrosine hydroxylase (TH), the rate‐limiting enzyme for DA biosynthesis (Molinoff and Axelrod 1971). To validate the use of MitoTimer as a tool to measure mitochondrial ROS generation in DA neurons, *Drosophila* were treated with H_2_O_2_, an established ROS generator. Live multiphoton imaging of ex vivo whole brains from both male and female flies (Figure 1A,B) revealed that H_2_O_2_ treatment significantly increased the MitoTimer red:green ratio in dopaminergic projections (Figure 1C,D). As further validation, TH‐driven MitoTimer flies were treated with 4‐hydroxy‐2,2,6,6‐tetramethylpiperidin‐1‐oxyl (TEMPOL), which metabolizes ROS (Wilcox 2010). Consistent with its antioxidant properties, TEMPOL decreased the MitoTimer red:green ratio in DA neuron projections across brain regions (Figure S1A,B). Just as importantly, concurrent treatment of flies with TEMPOL alongside H_2_O_2_ blocked the ability of H_2_O_2_ to significantly raise DA neuron mitochondrial ROS levels (Figure 1E,F). Together, these data demonstrate the feasibility of using MitoTimer to measure mitochondrial ROS in DA neurons in an ex vivo brain preparation.

### 2.2 Females Exhibit Lower Dopamine Neuron Mitochondrial Reactive Oxygen Species Levels

**FIGURE 2:**
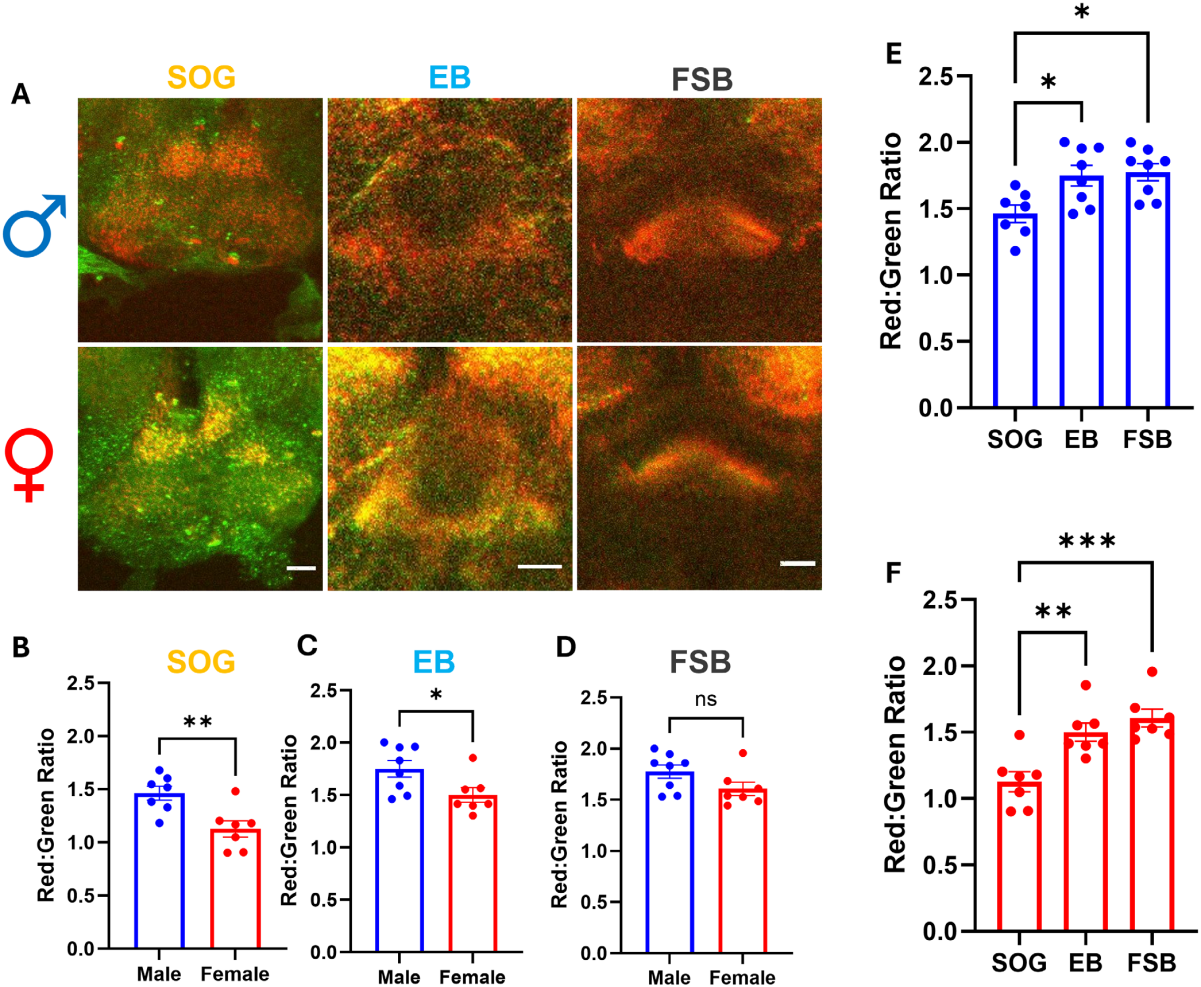
Sex‐ and region‐specific differences in basal levels of DA neuron mitochondrial ROS. (A) Representative images of MitoTimer‐labeled dopaminergic projections to the suboesophageal ganglion (SOG), ellipsoid body (EB), and fan‐shaped body (FSB) regions in adult brains of male (top) and female (bottom) control flies; images represent the overlap between red (oxidized) and green (unoxidized) MitoTimer fluorescence. Scale bars = 25 μm. (B–D) Quantification of MitoTimer red:green ratios revealed significantly lower baseline signal in females compared to males in DA neuron projections to the SOG (B; *t* = 2.709, *p* = 0.018, *n* = 7–8) and the EB (C; *t* = 2.378, *p* = 0.034, *n* = 7–8) but not to the FSB (D; *t* = 1.830, *p* = 0.090, *n* = 7–8). (E, F) Quantification of MitoTimer red:green ratios showed significantly lower basal ROS levels in the DA neuron projections to the SOG compared to both EB and FSB regions in males (E; *F*_(2,20)_ = 5.912, *p* = 0.010, *n* = 7–8) and females (*F*_(2,18)_ = 13.00, *p* = 0.0003, *n* = 7). Data are represented as means ± SEM. Student's unpaired *t*‐test (B–D); one‐way ANOVA with Tukey's multiple comparisons test (E, F). *p < 0.05; **p < 0.01; ***p < 0.001.

We next examined baseline levels of mitochondrial ROS within dopaminergic projections to three brain regions: the suboesophageal ganglion (SOG), the ellipsoid body (EB), and the fan‐shaped body (FSB) (Figure 2A). These regions receive dopaminergic projections from distinct sources, with the SOG receiving projections from local SOG DA neurons (Trisal et al. 2022) while the EB and FSB receive dopaminergic projections from the protocerebral posterior medial 3 (PPM3) cluster of DA neurons (Hartenstein et al. 2017; Mao and Davis 2009). Functionally, the FSB and EB are constituents of the central complex region that plays critical roles in navigation and adaptive behaviors, analogous to the mammalian basal ganglia (Hulse et al. 2021; Frighetto et al. 2022; Hu et al. 2018; Strausfeld and Hirth 2013). Females demonstrated significantly lower levels of basal mitochondrial ROS in dopaminergic projections to the SOG (20% lower; Figure 2B) and EB (14% lower; Figure 2C) but not to the FSB (Figure 2D) compared to males. We also discovered a significant difference in basal mitochondrial ROS in DA neuron projections across brain regions, with projections to the SOG having significantly lower ROS levels in both male (Figure 2E) and female (Figure 2F) flies compared to EB and FSB regions. Overall, we show important sex‐ and region‐specific differences in basal mitochondrial ROS levels within the brain DA system. These findings raise several questions: are there sex‐ and/or region‐specific differences in DA neuron mitochondrial ROS generation in response to physiological stressors such as aging or cell depolarization? Are there similar differences to pathological stressors like neurotoxicant exposure?

### 2.3 Aging Increases Mitochondrial Reactive Oxygen Species in Dopamine Neurons

**FIGURE 3:**
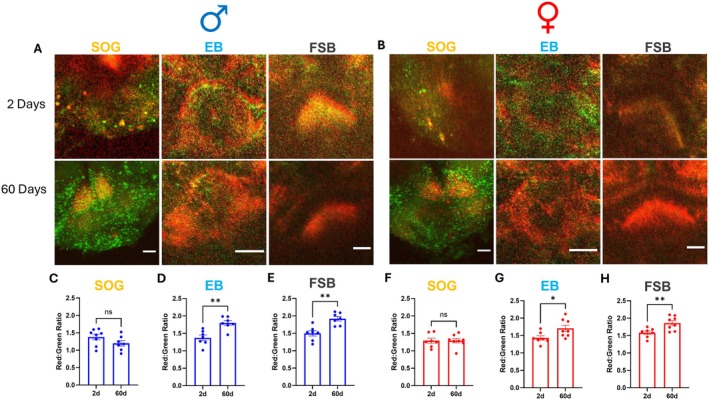
Aging increases DA neuron mitochondrial ROS in both males and females. (A, B) Representative images of MitoTimer‐labeled dopaminergic projections to the SOG, EB, and FSB regions in male (A) and female (B) control flies; images represent the overlap between red and green MitoTimer fluorescence. Scale bars = 25 μm. (C–E) Quantification of DA neuron‐specific MitoTimer red:green ratios revealed significantly higher levels of mitochondrial ROS in male flies at 60 days of age in the EB (D; *t* = 4.046, *p* = 0.002, *n* = 7) and FSB (E; *t =* 4.135, *p* = 0.001, *n* = 7) but not in the SOG (C; *t =* 1.584, *p* = 0.137, *n* = 7–8). (F–H) Quantification of MitoTimer red:green ratios demonstrated significantly higher levels of mitochondrial ROS in female dopaminergic projections to the EB (G; *t* = 2.448, *p* = 0.029, *n* = 7–8) and FSB (H; *t* = 3.077, *p* = 0.009, *n* = 7–8) but not to the SOG (F; *t* = 0.001, *p* = 0.999, *n* = 7–8). Data are represented as means ± SEM. Student's unpaired *t*‐test (C–H). *p < 0.05; **p < 0.01.

We next examined how aging impacts mitochondrial ROS levels in DA neurons. There is a well‐established connection between aging and the accumulation of mitochondrial ROS (Balaban et al. 2005; Giorgi et al. 2018). Previous work also showed sexually dimorphic DA neuron loss during healthy aging in *Drosophila*, with females more resilient than males to age‐related DA neurodegeneration (Buck et al. 2021a). We measured mitochondrial ROS in DA neurons of male (Figure 3A) and female (Figure 3B) flies at either 2‐ or 60‐days post‐eclosion (corresponding to young versus aged animals, respectively). Though aging did not alter mitochondrial ROS levels in DA neuron projections to the SOG (Figure 3C,F), projections to both the EB (males 31% increase; females 19% increase) and FSB (males 28% increase; females 17% increase) showed significantly increased ROS levels in aged flies compared to young flies (Figure 3D,E,G,H).

### 2.4 Paraquat Exposure Increases Mitochondrial Reactive Oxygen Species in a Sex‐Specific and dVGLUT‐Dependent Manner

**FIGURE 4:**
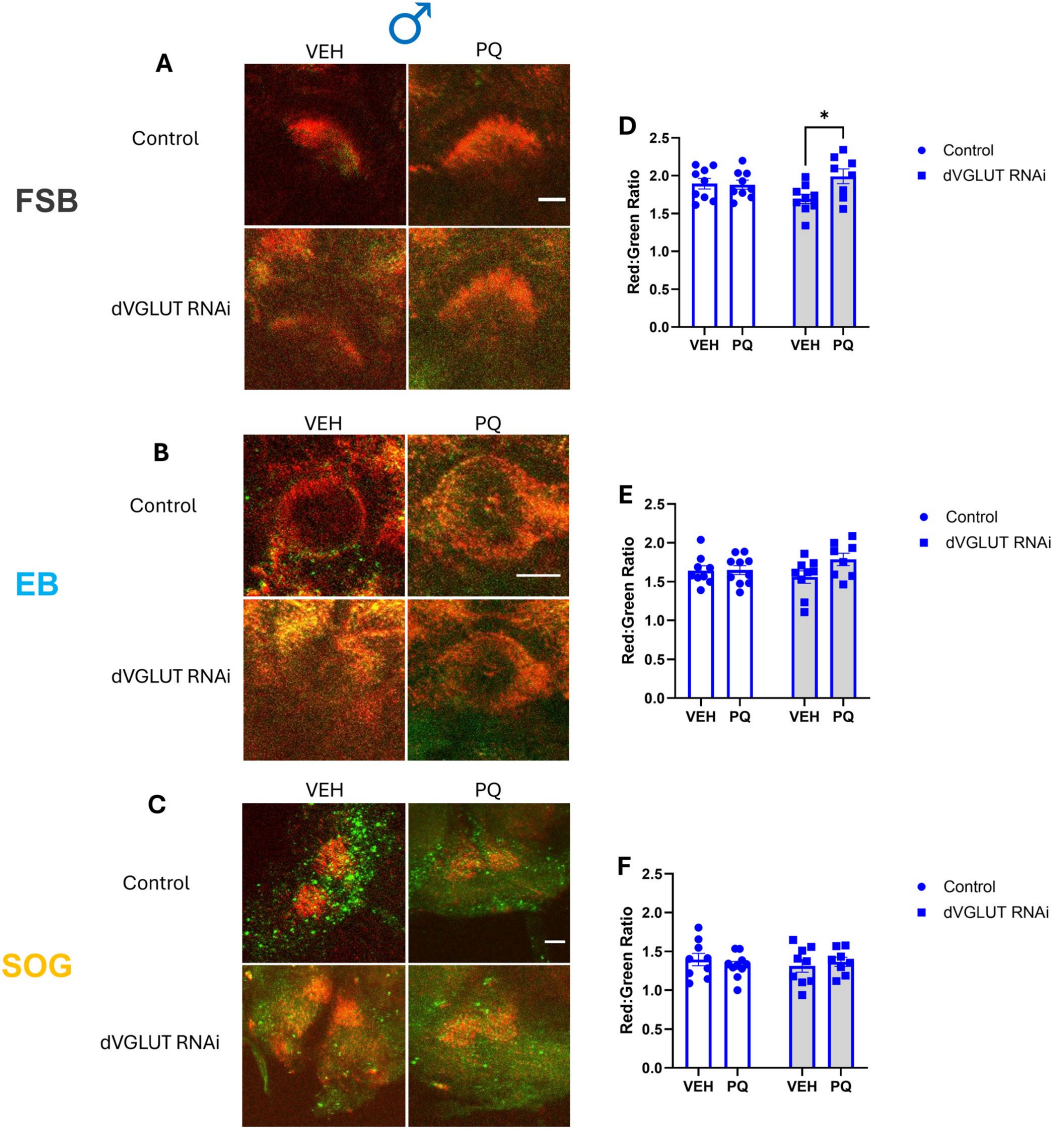
DA neuron‐specific dVGLUT knockdown selectively raises mitochondrial ROS in males following paraquat (PQ) exposure. (A–C) Representative images of MitoTimer‐labeled dopaminergic projections to the FSB, EB, and SOG in male DA neuron‐specific dVGLUT RNAi flies and controls (LexA RNAi). Scale bars = 25 μm. (D) Quantification of MitoTimer red:green ratios revealed significantly higher mitochondrial ROS levels within dopaminergic projections to the FSB of PQ‐treated (10 mM PQ, 10 days) dVGLUT RNAi male flies (*F*_(1,31)_ = 4.615, *p* = 0.040 for effect of interaction; *F*_(1,31)_ = 3.846, *p* = 0.059 for effect of PQ, *F*_(1,31)_ = 0.391, *p* = 0.536 for effect of genotype, *n* = 8–9). (E) Quantification of MitoTimer red:green ratios demonstrated no significant differences in DA neuron mitochondrial ROS levels in the EB after PQ treatment versus the vehicle control (*F*_(1,32)_ = 2.445, *p* = 0.128 for effect of interaction; *F*_(1,32)_ = 2.773, *p* = 0.106 for effect of PQ, *F*_(1,32)_ = 0.148, *p* = 0.703 for effect of genotype, *n* = 8–9). (F) Quantification of MitoTimer red:green ratios demonstrated no difference in DA neuron mitochondrial ROS levels after PQ treatment compared to the vehicle control (*F*_(1,32)_ = 0.865, *p* = 0.359, for effect of interaction; *F*_(1,32)_ = 0.028, *p* = 0.868 for effect of PQ; *F*_(1,32)_ = 0.067, *p* = 0.797 for effect of genotype, *n* = 8–9). Data are represented as means ± SEM. Two‐way ANOVA (E, F) with Tukey's multiple comparisons test (D). *p < 0.05.

In addition to its roles in aging, we and others have shown that expression of VGLUT2 and dVGLUT play key roles in DA neuron resilience in response to insults, including in mammalian PD models and in clinical PD (Steinkellner et al. 2022; Shen et al. 2018; Buck et al. 2021a, 2022, 2021b). Thus, we determined whether a mechanism for dVGLUT/VGLUT2‐mediated resiliency is through the transporter's ability to modulate mitochondrial ROS in response to insults. We selected the paraquat (PQ) model of PD (Aryal and Lee 2019) as it is a widely used pesticide (Goldman 2014) whose exposure increases PD risk in humans (Tanner et al. 2009). PQ is therefore used to model toxicant‐induced DA neurodegeneration in rodents and flies (Lal et al. 2024; See et al. 2022; Sharma and Mittal 2024). We concurrently expressed MitoTimer and knocked down dVGLUT expression specifically in DA neurons using a previously validated dVGLUT RNA interference (RNAi) strain (Buck et al. 2021a; Aguilar et al. 2017). Following 10 days of PQ exposure, we ascertained the impact of diminished DA neuron‐specific dVGLUT expression on PQ‐induced DA neuron mitochondrial ROS accumulation (Figure 4A–C). We identified a PQ‐induced increase in mitochondrial ROS specifically in male dopaminergic projections to the FSB of dVGLUT RNAi animals (18% increase, Figure 4D). However, this PQ effect was absent in projections to both the EB (Figure 4E) and SOG (Figure 4F). In contrast, female flies (Figure S2A–C) showed no effects of PQ in all three brain regions, including in the presence of dVGLUT RNAi (Figure S2D–F). These data align with the wealth of evidence demonstrating males' higher susceptibility to DA neurodegeneration in preclinical studies (De Miranda et al. 2019) and clinically in humans (Ben‐Shlomo et al. 2024). Collectively, our results suggest a role for dVGLUT in mediating susceptibility to cytotoxic ROS accumulation particularly in males, which may contribute to the sex differences in DA neuron vulnerability in PD.

### 2.5 DA Neuron dVGLUT Is a Modulator of Depolarization‐Induced Increases in Intracellular ATP

**FIGURE 5:**
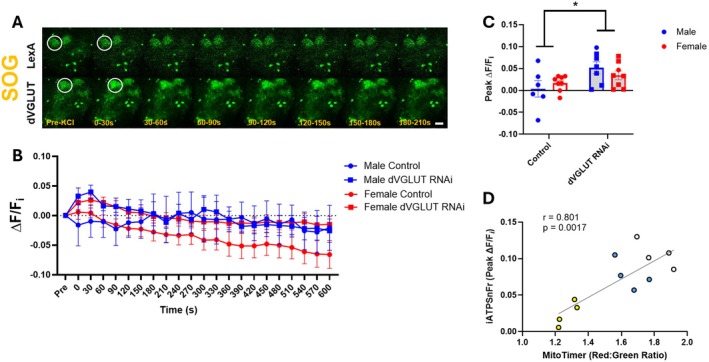
dVGLUT modulates region‐specific, activity‐driven increases in intracellular ATP in dopaminergic projections. (A) Representative images of dopaminergic projections to the SOG labeled by iATPSnFr, an intracellular ATP reporter, in DA neuron‐specific dVGLUT RNAi and control (LexA RNAi) flies. Scale bars = 25 μm. (B) Quantification of iATPSnFr fluorescence in DA neuron projections to the SOG before and after KCl‐induced depolarization. (C) Quantification of peak iATPSnFr Δ*F*/*F*_i_ in the SOG revealed increased activity‐driven intracellular ATP accumulation in DA neurons of dVGLUT RNAi flies (*F*_(1,25)_ = 1.576, *p* = 0.221 for effect of interaction, *F*_(1,256)_ = 7.41, *p* = 0.012 for effect of genotype, *F*_(1,25)_ = 0.00003, *p* = 0.873, *n* = 6–8). (D) Baseline MitoTimer red:green ratio and iATPSnFr peak ΔF/F_i_ after KCl‐induced depolarization across the SOG (yellow), EB (blue), and FSB (white) were strongly correlated (*r* = 8.01, *p* = 0.002, *n* = 12 with 4 per region). Data are represented as means ± SEM. Two‐way ANOVA with Tukey's multiple comparisons test (C); Pearson's correlation (D). *p < 0.05.

We further explored the relationships between dVGLUT, activity‐driven ATP synthesis, and mitochondrial ROS generation since mitochondrial ATP synthesis inherently generates ROS during electron transport between respiratory complexes (Kowalczyk et al. 2021; Shadel and Horvath 2015). Neuronal depolarization contributes to ROS generation by placing stress on the respiratory chain to generate more ATP to fuel such a metabolically demanding state (Murphy 2009; van Hameren et al. 2019; Ackermann et al. 2024). We posited that dVGLUT's effects on mitochondrial ROS levels are due to its ability to modulate mitochondrial ATP production, particularly during states of depolarization‐induced stress. To test this, we measured the impact of KCl‐induced depolarization on DA neuron ATP production via TH‐driven iATPSnFR, a genetically‐encoded fluorescent reporter of intracellular ATP (Lobas et al. 2019), in the presence or absence of DA neuron‐specific dVGLUT knockdown (Figure 5A, Figure S3). To validate the use of the iATPSnFR reporter for measuring cytosolic ATP levels in DA neurons, we demonstrated that exogenous ATP perfusion significantly increased peak iATPSnFR signal in dopaminergic projections to the SOG (Figure S3A,B). In both males and females, DA neuron‐specific dVGLUT knockdown increased peak iATPSnFR signal (ΔF/F_i_) in dopaminergic projections to the SOG (198% increase, Figure 5B,C) but not to the EB (Figure S3C,D) or FSB (Figure S3E,F). This indicates that dVGLUT's impact on depolarization‐induced DA neuron ATP synthesis is region‐specific. Strikingly, the MitoTimer red:green ratio and peak iATPSnFR ΔF/F_i_ were strongly correlated (Figure 5D), further connecting activity‐driven ATP synthesis and mitochondrial ROS in DA neurons. These data suggest that dVGLUT is a physiological modulator of activity‐dependent generation of ATP. Interestingly, the sex differences observed in the above MitoTimer studies (Figure 4) were absent in our iATPSnFR‐based studies, pointing to a potential sex difference in how DA neurons handle mitochondrial ROS despite similar levels of ATP synthesis during periods of cell depolarization.

### 2.6 dVGLUT Modulates Depolarization‐Induced Increases in Mitochondrial Reactive Oxygen Species in Dopamine Neurons

**FIGURE 6:**
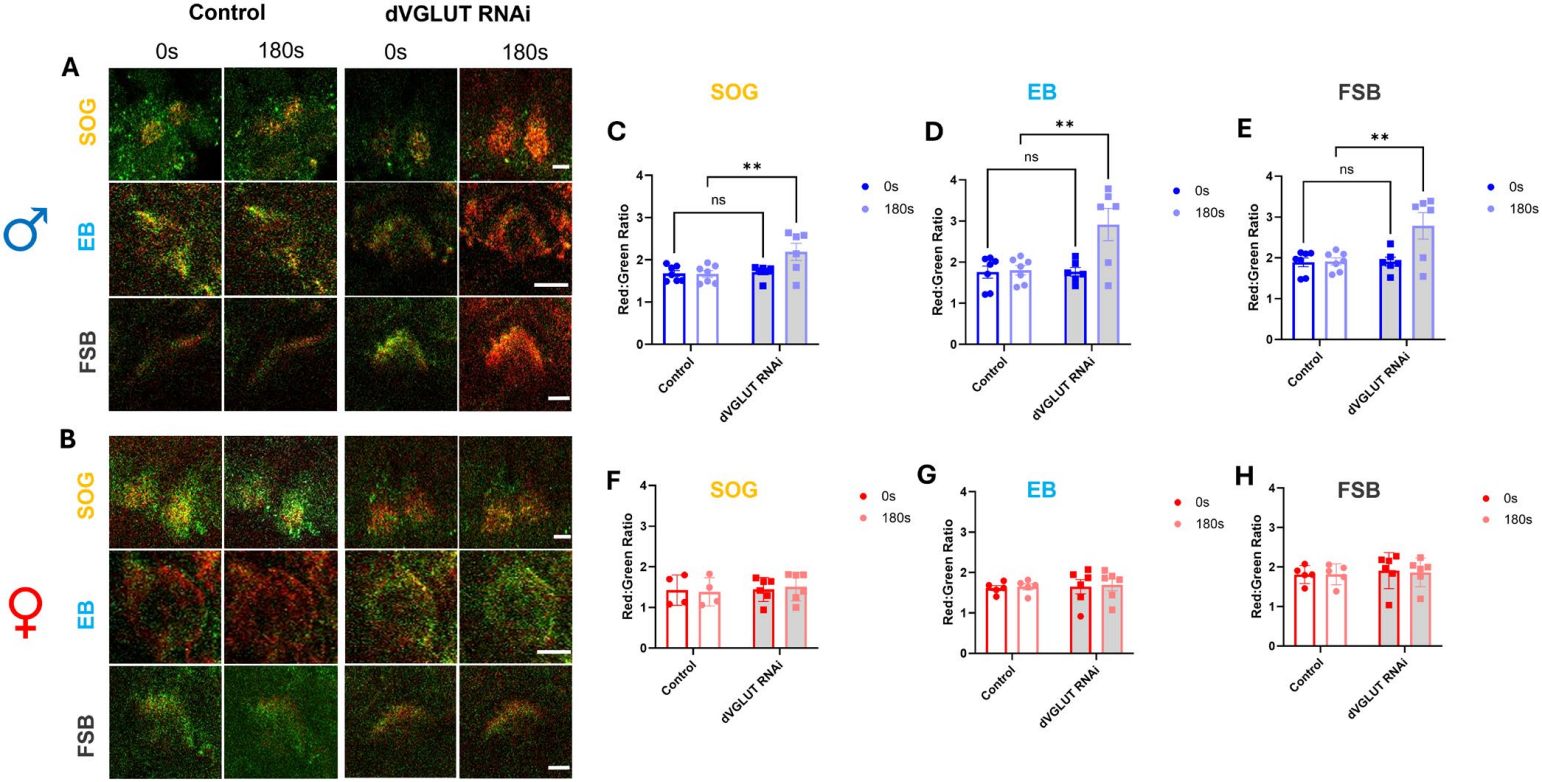
Depolarization increases DA neuron mitochondrial ROS in a dVGLUT‐dependent manner in males. (A, B) Representative images of MitoTimer labeled dopaminergic projections to the SOG, EB, and FSB in DA neuron‐specific dVGLUT RNAi and control (LexA RNAi) flies. Scale bars = 25 μm. (C–E) Quantification of MitoTimer red:green ratios revealed significantly higher depolarization‐induced increases in mitochondrial ROS levels in DA neuron projections of male dVGLUT RNAi flies in the SOG (C; *F*_(1,11)_ = 11.02, *p* = 0.007 for effect of interaction; *F*_(1,11)_ = 3.778, *p* = 0.078 for effect of genotype; *F*_(1,11)_ = 10.05, *p* = 0.009 for effect of time, *n* = 6–7), the EB (D; *F*_(1,11)_ = 11.87, *p* = 0.006 for effect of interaction; *F*_(1,11)_ = 4.817, *p* = 0.051 for effect of genotype; *F*_(1,11)_ = 13.90, *p* = 0.003 for effect of time, *n* = 6–7) and the FSB (E; *F*_(1,11)_ = 11.71, *p* = 0.006 for effect of interaction; *F*_(1,11)_ = 4.305, *p* = 0.062 for effect of genotype; *F*_(1,11)_ = 12.96, *p* 0.004 for effect of time, *n* = 6–7). (F–H) Quantification of MitoTimer red:green ratios demonstrated no differences in depolarization‐induced mitochondrial ROS levels in female flies in the SOG (F; *F*_(1,8)_ = 2.927, *p* = 0.126 for effect of interaction; *F*_(1,8)_ = 0.107, *p* = 0.752 for effect of genotype; *F*_(1,8)_ = 0.118, *p* = 0.740 for effect of time, *n* = 4–6) the EB (G; *F*_(1,9)_ = 0.056, *p* = 0.819 for effect of interaction; *F*_(1,9)_ = 0.048, *p* = 0.831 for effect of genotype; *F*_(1,9)_ = 2.412, *p* = 0.155 for effect of time, *n* = 5–6) and the FSB (H; *F*_(1,9)_ = 0.441, *p* = 0.523 for effect of interaction; *F*_(1,9)_ = 0.129, *p* = 0.728 for effect of genotype; *F*_(1,9)_ = 0.292, *p* = 0.602 for effect of time, *n* = 5–6). Data are represented as means ± SEM. Repeated measured two‐way ANOVA (F–H) with Sidak's multiple comparisons test (C–E). **p < 0.01.

Finally, since DA neuron dVGLUT modulates mitochondrial ROS in the setting of PQ exposure (Figure 4), we asked whether dVGLUT expression similarly impacts mitochondrial ROS in the context of neuronal depolarization—a physiological driver of transient metabolic stress (Auten and Davis 2009). TH‐driven MitoTimer was imaged in real time in male and female DA neurons while undergoing KCl‐induced depolarization (Figure 6A,B). Male DA neuron‐specific dVGLUT RNAi flies demonstrated a depolarization‐induced increase in mitochondrial ROS in dopaminergic projections to all three brain regions (SOG 28% increase; EB 65% increase; FSB 46% increase). These depolarization‐induced alterations in ROS were absent in control flies (Figure 6C–E). Our results mirror the changes in intracellular ATP levels in dopaminergic projections to the SOG (Figure 5C) and strongly suggest that males require a reserve of dVGLUT in DA neurons to adequately guard against activity‐dependent increases in cytotoxic mitochondrial ROS. Females with DA neuron‐specific dVGLUT knockdown did not demonstrate a significant change in the MitoTimer red:green ratio in response to depolarization irrespective of dVGLUT knockdown (Figure 6F–H). In contrast to males, our data suggest that female DA neurons likely harbor additional protective mechanisms to buffer against excessive mitochondrial ROS accumulation in the setting of context‐dependent increases in metabolic demand.

## 3 Discussion

In this study, we showed a fundamental sex difference in DA neuron mitochondrial ROS levels at baseline and similar sexual dimorphism in acute ROS generation in response to various stressors, including aging and PQ exposure. We demonstrated that females have lower mitochondrial ROS across multiple brain regions. Aging increased mitochondrial ROS in both sexes except in the SOG, the region of the brain that showed the lowest baseline ROS levels. PQ, a common model of PD in *Drosophila* (Aryal and Lee 2019), selectively increased ROS in dopaminergic projections to the FSB of male flies in the presence of dVGLUT knockdown. This agrees with recent data demonstrating the protective nature of dVGLUT/VGLUT2 in fly and mammalian DA neurons (Steinkellner et al. 2018; Shen et al. 2018; Buck et al. 2021a). We also discovered an activity‐dependent relationship between DA neuron dVGLUT expression and intracellular ATP generation in both sexes. However, examination of depolarization‐induced changes in DA neuron mitochondrial ROS revealed that dVGLUT knockdown made males selectively more vulnerable to ROS accumulation compared to females. Together, these data demonstrate a unique sex difference in the capacity of DA neurons to deal with ROS during both energetically demanding states and in response to toxicant insults.

We identified a regional difference in baseline mitochondrial ROS levels in DA neurons. Dopaminergic projections to the SOG demonstrated significantly lower ROS compared to projections to the EB and FSB. There is a growing understanding of the protective role of dVGLUT and its mammalian ortholog VGLUT2 in DA neurodegeneration both in models of PD and aging (Steinkellner et al. 2018, 2022; Shen et al. 2018; Buck et al. 2021a, 2022). In accordance with this idea, we also demonstrated the absence of age‐related changes in accumulated mitochondrial ROS specifically in dopaminergic projections to the SOG in both sexes, despite seeing significant increases in projections to the EB and FSB. We therefore speculate that the intrinsically lower levels of ROS in SOG projections are due to the high percentage of dopaminergic neurons that express dVGLUT and project to this brain region (Buck et al. 2023).

Dopaminergic cells that co‐express VGLUT2 are more likely to survive in mammalian models of PD (Steinkellner et al. 2018; Shen et al. 2018; De Miranda et al. 2019) and this same subset of DA neurons is more resilient in human PD patients as well (Steinkellner et al. 2022). To model PD in *Drosophila*, we exposed them to PQ (Aryal and Lee 2019), a widely used pesticide (Goldman 2014) whose exposure also increases PD risk in humans (Tanner et al. 2009). Ultimately, we showed that DA neuron dVGLUT knockdown increased mitochondrial ROS accumulation specifically in PQ‐exposed males—an effect that was absent in females. Importantly, in humans, PD is more commonly diagnosed in men than women (Ben‐Shlomo et al. 2024), and recent evidence suggests that females express more VGLUT2 in DA neurons than males in humans, rodents, and flies (Buck et al. 2021a). Females are more resilient to DA neurodegeneration in mammalian PD models as well (De Miranda et al. 2019; Dluzen et al. 1996), which we attribute to their higher numbers of VGLUT2‐expressing DA neurons. Consistent with these data, we also demonstrated that females have reduced mitochondrial ROS at baseline across brain regions. This raises the possibility that higher dVGLUT expression in females is a factor driving the differences both at baseline and in our model of PD (Buck et al. 2021a).

We found that dVGLUT knockdown increases both intracellular ATP levels and mitochondrial ROS in DA neurons during depolarization. ATP production directly leads to accumulation of mitochondrial ROS in axons (van Hameren et al. 2019; Ackermann et al. 2024), which is consistent with our results. Notably, while loss of dVGLUT caused increased accumulation of ATP in response to neuronal depolarization in both sexes, the parallel effect on mitochondrial ROS was male‐specific. This suggests that females may possess additional mechanisms to buffer depolarization‐induced mitochondrial ROS production that are dVGLUT‐independent and are sex‐specific. Additionally, we uncovered a regional correlation between baseline mitochondrial ROS and intracellular ATP during depolarization in DA neurons. The SOG, which has the highest levels of dVGLUT‐expressing DA neuron projections (Buck et al. 2023), has the lowest levels of baseline ROS as well as the lowest levels of activity‐dependent intracellular ATP accumulation. These data are consistent with recent studies in mice and humans showing that VTA neurons, which are enriched in VGLUT2‐expressing DA neurons (Steinkellner et al. 2018, 2022; Poulin et al. 2018; Yamaguchi et al. 2015; Buck et al. 2025), are more resilient to DA neurodegeneration (Steinkellner et al. 2018, 2022; Shen et al. 2018). Moreover, these VTA cells have lower baseline mitochondrial ROS and lower baseline ATP production compared to other midbrain dopaminergic populations (Pacelli et al. 2015).

We identified region‐specific differences in both mitochondrial ROS and cytoplasmic ATP accumulation within DA neurons across aging and in our model of PD. While the SOG was protected against aging and PQ‐induced increases in ROS, male dVGLUT RNAi flies demonstrated significantly higher ROS in the SOG after depolarization. We posit that this discrepancy between DA neuron resilience and ROS levels specifically in the SOG DA neurons may be related to additional dVGLUT‐independent mechanisms. It is therefore possible that SOG neurons, beyond their higher levels of dVGLUT expression, have some additional capacity to deal with age‐related insults that is separate from their ability to resist depolarization‐induced increases in ROS. Consistent with this, while aging impacts cell physiology and transcriptional profiles, subsets of *Drosophila* neurons, including within the SOG, remain resilient through old age (Davie et al. 2018), providing an ideal model system with which to elucidate both dVGLUT‐dependent and ‐independent mechanisms. Future follow‐up studies will examine how ROS and ATP generation in DA neurons are modulated across different brain regions in the context of aging with and without dVGLUT knockdown.

How does dVGLUT/VGLUT2 expression confer DA neuroprotection? We previously demonstrated that dVGLUT/VGLUT2 enhances vesicular DA loading by mediating activity‐dependent vesicle hyperacidification (Aguilar et al. 2017). dVGLUT/VGLUT2‐mediated enhanced DA loading reduces cytosolic DA, which diminishes the overall oxidative burden of the cell as cytosolic DA is readily oxidizable and can be damaging to cell health (Hastings et al. 1996; Zhang et al. 2019). Finally, VGLUTs were originally characterized as phosphate transporters and demonstrate phosphate transporter activity when on the cell surface (Preobraschenski et al. 2018), offering an additional alternative mechanism for potentially modulating intracellular ATP levels and subsequently ROS. It is also possible that co‐transmitting DA/glutamate neurons have enhanced neuroprotection via alternative mechanisms not directly related to dVGLUT/VGLUT2. For example, increased levels of glutamate in these cells can contribute to increased glutathione (GSH) biosynthesis (Sedlak et al. 2019; Franco and Cidlowski 2009). Increased levels of GSH could directly contribute to DA neurons' capabilities of buffering ROS, as GSH can, either directly or in conjunction with other enzymes, reduce ROS (Dringen 2000; Smeyne and Smeyne 2013). There is evidence of decreased GSH in preclinical PD models and the SNc of human PD patients (Smeyne and Smeyne 2013; Wüllner et al. 1996; Abdel‐Salam 2008; Pearce et al. 1997), indicating a role for GSH and its ability to reduce ROS in the resiliency of DA neurons that also co‐express glutamatergic machinery.

Although differences in DA neuron dVGLUT expression represent one of the main driving factors in the sex‐ and region‐specific differences in mitochondrial ROS, further experiments are required to better understand the precise mechanisms for the increased ROS protection in females. We posit that the higher baseline level of dVGLUT in female DA neurons (Buck et al. 2021a) can compensate for at least some of the dVGLUT knockdown in our RNAi studies to still confer resilience to DA neurons. To address this, future work will employ CRISPR‐Cas9 technology to create a complete knockout (KO) of dVGLUT in DA neurons in female flies. These complete DA neuron‐specific dVGLUT KO flies will then be examined in the contexts of aging and PD. It is also possible that females possess additional dVGLUT/VGLUT2‐independent mechanisms of protection that confer DA neuron resiliency. Indeed, the male‐specific *Sex determining region of the Y chromosome* (*SRY*) gene is upregulated in animal PD models, and SRY inhibition protects against DA neurodegeneration (Lee et al. 2019). Additionally, previous reports indicate a neuroprotective role of estrogen (Adamson et al. 2022) as well as higher baseline expression of antioxidant enzymes in DA neurons, which may further explain greater DA neuron resilience in females in multiple PD models (Kenchappa et al. 2004; Ciron et al. 2015). Subsequent work will address the impacts of sex hormones and *SRY* expression within the context of dVGLUT/VGLUT2‐mediated DA neuroprotection in aging and PD.

In conclusion, our findings demonstrate a sex difference in mitochondrial ROS in DA neurons. There is a clear interplay between DA neuron vulnerability to cell stress and dVGLUT expression, including a notable sexual dimorphism in DA neuron resilience. Together, our data contribute to a growing body of evidence demonstrating sex differences in DA neuron vulnerability and the role of dVGLUT in DA neuron resiliency. Defining these mechanisms of DA neuron resilience in females versus males may ultimately lead to more effective PD treatments in both men and women.

## 4 Materials and Methods

### 4.1 *Drosophila* Rearing, Stocks and Drug Treatments

All *Drosophila melanogaster* stocks were maintained on standard cornmeal‐molasses fly food at 24°C with 60%–70% humidity under a 12:12 light–dark schedule. All fly stocks were obtained from the Bloomington *Drosophila* Stock Center (BDSC) unless otherwise noted. *Drosophila* stocks include TH‐GAL4 (BDSC #8848), UAS‐LexA RNAi (BDSC #67946), UAS‐Luciferase (BDSC #35788), UAS‐dVGLUT RNAi (BDSC #40845), and UAS‐iATPSnFr (Gift from Drs. Kevin Mann and Thomas Clandinin) (Mann et al. 2021). Adult flies were aged 14 days post‐eclosion, unless reported otherwise. Final genotypes tested include: (1) TH‐GAL4, UAS‐MitoTimer/UAS‐LexA RNAi. (2) TH‐GAL4, UAS‐MitoTimer/UAS‐dVGLUT RNAi. (3) TH‐GAL4, UAS‐MitoTimer/UAS‐Luciferase. (4) UAS‐iATPSnFR/+; TH‐GAL4/UAS‐LexA RNAi. (5) UAS‐iATPSnFR/+;TH‐GAL4/UAS‐dVGLUT RNAi. For all figures except for Figure 2, flies expressing UAS‐LexA RNAi were used as the control flies. For Figure 2, flies expressing UAS‐Luciferase were used as the respective control group.

All drugs were purchased from Sigma‐Aldrich (St. Louis, MO). Drugs were diluted to the final concentrations in melted standard cornmeal‐molasses fly food: paraquat dichloride (PQ; 10 mM), hydrogen peroxide (H_2_O_2_; 3%) and 4‐hydroxy‐2,2,6,6,‐tetramethylpiperidin‐1‐oxyl (TEMPOL; 3 mM).

### 4.2 Ex Vivo Multiphoton *Drosophila* Brain Imaging

#### 4.2.1 Dissection and Microscopy

Whole adult *Drosophila* brains were isolated by brain microdissection and removed in Schneider's *Drosophila* Medium (Gibco, Waltham, MA). Brains were immobilized via pinning with tungsten wire (California Fine Wire Company, Grover Beach, CA) onto Sylgard dishes as described previously (Aguilar et al. 2017; Freyberg et al. 2016). Brains were subsequently bathed in adult hemolymph‐like saline (AHL; 108 mM NaCl, 5 mM KCl, 2 mM CaCl_2_, 8.2 mM MgCl_2_, 1 mM NaH_2_PO_4_, 10 mM sucrose, 5 mM trehalose, 5 mM HEPES, 4 mM NaHCO_3_; pH 7.4, 265 mOsm) for imaging, as previously described (Freyberg et al. 2016). For KCl‐induced depolarization, we employed isosmotic high KCl AHL (78 mM NaCl, 40 mM KCl, 2 mM CaCl_2_, 8.2 mM MgCl_2_, 1 mM NaH_2_PO_4_, 10 mM sucrose, 5 mM trehalose, 5 mM HEPES, 4 mM NaHCO_3_; pH 7.4, 265 mOsm) which was continuously perfused (flow rate: 2 mL/min) over the brains during imaging using a Watson‐Marlow 120S peristaltic pump (Watson‐Marlow, Cornwall, UK).

#### 4.2.2 Brain Imaging

Ex vivo brain preparations were imaged on a Bergamo II resonant‐scanning two‐photon microscope (Thorlabs, Newton, NJ) using a 20× (1.0 NA) water immersion objective lens (Olympus) and an Insight X3 laser (Spectra‐Physics Inc., Milpitas, CA). Less than 50 mW mean power was delivered through the objective for all experiments. Gain and Pockels cell settings for power were fixed for all imaging experiments. For the imaging of iATPSnFr and unoxidized MitoTimer biosensors, the tunable laser output was mode‐locked at 920 nm. These fluorescent emissions were collected using a 525/50 nm full‐width half‐maximum (FWHM) bandpass emission filter. For imaging of oxidized MitoTimer, the laser was fixed at 1045 nm. Fluorescent emissions were collected using a 607/70 nm FWHM bandpass emission filter. Imaging parameters were optimized to avoid pixel saturation. Data acquisition was performed with ThorImage software (Versions 4.0/4.1; Thorlabs).

#### 4.2.3 MitoTimer Data Acquisition

For single time point experiments, z‐stacks of optical sections were acquired through the entire depth of the fly brain in 2 μm z‐steps with 8× frame averaging. For multi‐timepoint experiments with KCl, the same 2 μm z‐steps were used but without frame averaging. Single images were 16‐bit, 1024 × 1024 pixels (pixel size of 0.64 μm) except for KCl experimental data, which were collected as 16‐bit, 512 × 512 pixels (pixel size 1.28 μm) images.

#### 4.2.4 iATPSnFr Data Acquisition

All iATPSnFr experiments were collected as z‐stacks over time in the presence of KCl as 16‐bit images at 512 × 512 pixels. These images were collected in 3 μm z‐steps with no frame averaging.

### 4.3 Image Analysis

Average z‐projections of the image stacks were made using Fiji/ImageJ (National Institutes of Health, Bethesda, MD). MitoTimer‐labeled and iATPSnFr‐labeled dopaminergic projections were analyzed throughout z‐stacks in both male and female *Drosophila*.

#### 4.3.1 MitoTimer

Mean pixel fluorescence intensity projections from both the green (unoxidized) and red (oxidized) emission channels were created for the SOG, EB, and FSB brain regions. MitoTimer oxidation levels were expressed as the ratio of red to green fluorescence (red:green ratio). Mean pixel fluorescence intensities of both the red and green channels were quantified across brain regions and across time points.

#### 4.3.2 iATPSnFr

Mean pixel fluorescence intensity projections were made from the green fluorescence emission channel for the SOG, EB, and FSB brain regions. The mean pixel fluorescent intensity value pre‐KCl (*F*_i_) was subtracted from each subsequent post‐KCl timepoint (F) and then divided by *F*_i_ to quantify the change in iATPSnFr fluorescence over time in response to KCl. This measurement (*F*‐ *F*_i_)/*F*_i_ is abbreviated as ∆*F*/*F*.

### 4.4 Statistical Analyses

Experiments were designed after power calculations based on preliminary data using G*Power 3.1 software (Faul et al. 2009) (Heinrich‐Heine‐Universität Düsseldorf, Düsseldorf, Germany). All other statistical analyses were run on GraphPad Prism 10.1 (GraphPad Software, San Diego, CA). Shapiro–Wilk and Kolmogorov–Smirnov normality tests were performed on all data to ensure they were normally distributed, and Bartlett's tests were performed to check for heteroscedasticity. For all comparisons between two groups, unpaired t‐tests were used. For both one‐way and two‐way analysis of variance (ANOVAs), Tukey's multiple comparisons post hoc was used. For all repeated measures ANOVAs, Sidak's multiple comparisons post hoc was used. Pearson's correlation coefficient was used for all correlations. For all experiments, *p < 0.05, **p < 0.01, and ***p < 0.001.

## Author Contributions

S.A.B. and Z.F. conceived the project. S.A.B., S.J.M., T.K., Z.Y., S.A.R., J.Y., C.E.J.C., and Z.F. performed imaging experiments and data analysis. S.A.B., S.J.M., and Z.F. wrote the manuscript with contributions from co‐authors.

## Conflicts of Interest

Z.F. is funded by an investigator‐initiated award from UPMC Enterprises. The remaining authors declare no conflicts of interest.

## Supporting information


Data S1.

**Appendix S1.** Supporting Information.

## Data Availability

The data that support the findings of this study are available on request from the corresponding author.
