# Supplementary material for: dVGLUT Is a Mediator of Sex Differences in Dopamine Neuron Mitochondrial Function Across Aging and in a Parkinson's Disease Model
Source: Aging Cell. 2025 May 12;24(8):e70096. doi: 10.1111/acel.70096 (PMC12341811; doi:10.1111/acel.70096)
Supplement: Supplementary file 1 — Data S1. [file ACEL-24-e70096-s001.pdf]

Supplementary Information

dVGLUT mediates sex differences in dopamine neuron mitochondrial function across aging and in a Parkinson's disease model

Silas A. Buck[^], Samuel J. Mabry[^], Tenzin Kunkhyen, Zilu Yang, Sophie A. Rubin, Jinting Yang, Claire E. J. Cheetham, Zachary Freyberg[*]

[^]Co-first authors

[*]Lead contact: Zachary Freyberg, M.D., Ph.D. (freyberg@pitt.edu)

Figures S1-S3

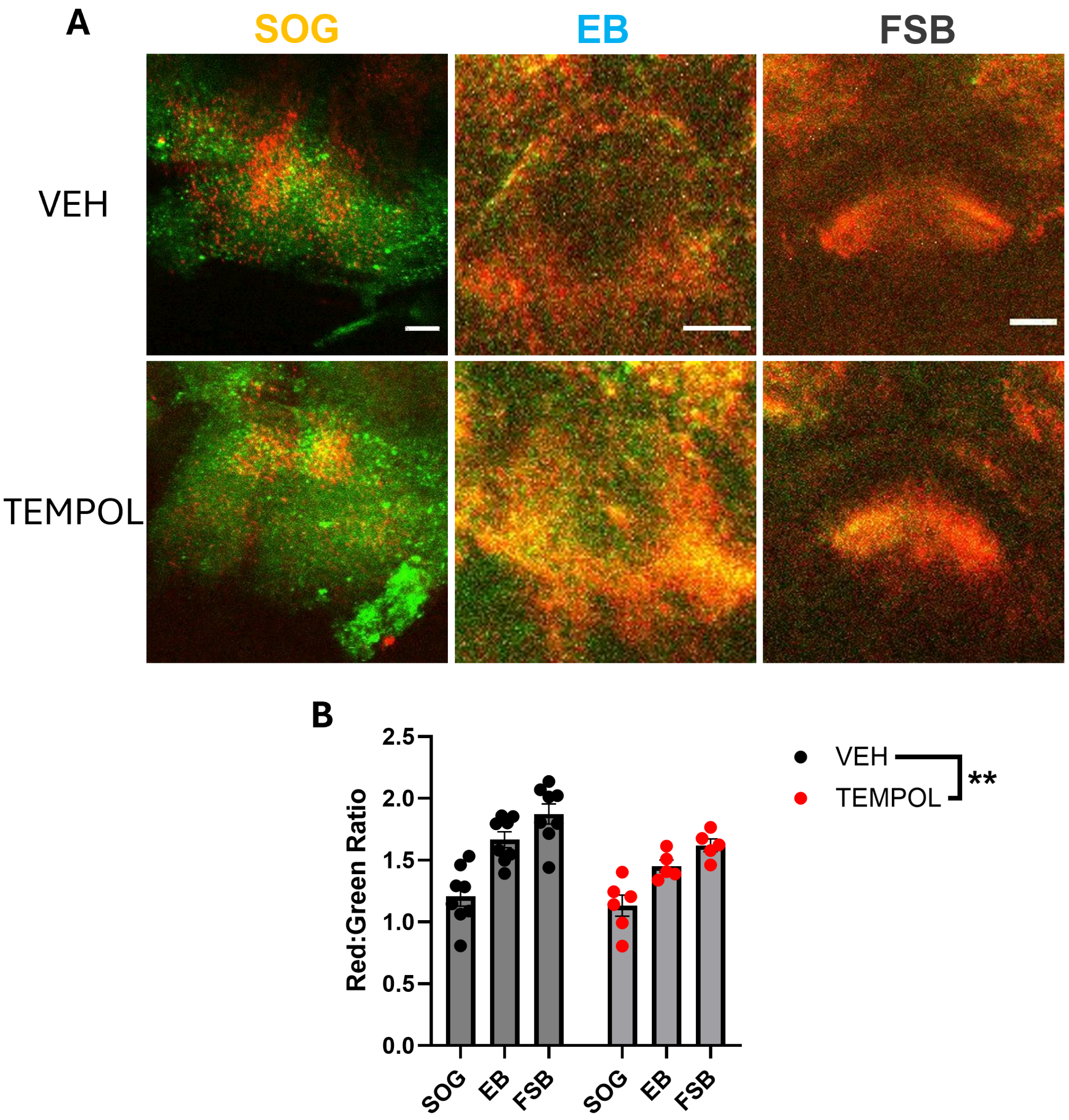

Figure S1. TEMPOL treatment reduces mitochondrial ROS and blocks the effects of H_2O_2 in DA neurons. (A) Representative images of MitoTimer-labeled dopaminergic projections to the SOG, EB, and FSB in male control flies (LexA RNAi). Flies were exposed to either TEMPOL (3mM, 5d) or the vehicle control (VEH). Scale bars = 25µm. **(B)** Quantification of MitoTimer red:green ratios in dopaminergic projections to all 3 brain regions revealed a significant decrease in signal in response to TEMPOL treatment compared to vehicle ($F_{(1, 34)} = 8.251$, $p = 0.007$, n = 5-8 for effect of TEMPOL; $F_{(2, 34)} = 29.55$, $p < 0.001$; $F_{(2, 34)} = 0.734$, $p = 0.487$ for effect of interaction, n = 5-8). Data are represented as means ± SEM. Two-way ANOVA **(B)**. $^{**}p < 0.01$.

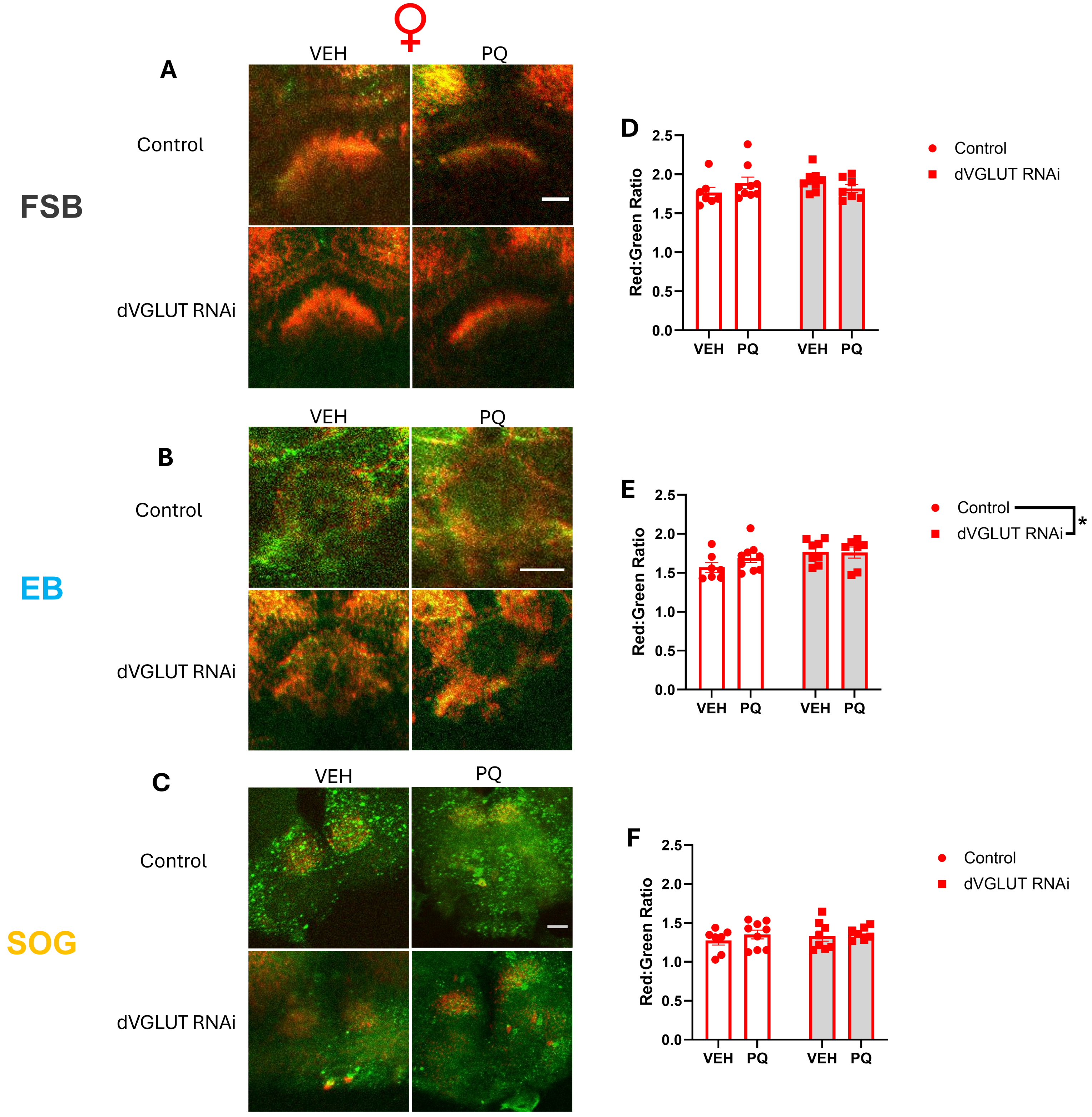

♀
A
B
C
VEH
PQ
Control
dVGLUT RNAi
FSB
EB
SOG
D
E
F
Red:Green Ratio
2.5
2.0
1.5
1.0
0.5
0.0
VEH
PQ
VEH
PQ
Control
dVGLUT RNAi
*

Figure S2. PQ exposure does not increase DA neuron mitochondrial ROS in females. (A-C) Representative images of MitoTimer-labeled dopaminergic projections to the FSB, EB, and SOG regions in adult female dVGLUT RNAi flies and LexA RNAi controls; images represent the overlap between red and green MitoTimer fluorescence. Scale bars = 25μm. **(D)** Quantification of MitoTimer red:green ratios demonstrated no significant difference in mitochondrial ROS levels in DA neuron projections to the FSB after PQ treatment ($F_{(1, 27)} = 3.031$, $p = 0.093$ for effect of interaction; $F_{(1, 27)} = 0.038$, $p = 0.846$ for effect of PQ, $F_{(1, 27)} = 0.383$, $p = 0.541$ for effect of genotype, $n = 7\text{-}9$). **(E)** Despite no significant PQ-induced alterations in mitochondrial ROS levels in dopaminergic projections to the EB, female dVGLUT RNAi flies demonstrated an overall increase in ROS irrespective of treatment ($F_{(1, 27)} = 1.149$, $p = 0.29$ for effect of interaction; $F_{(1, 27)} = 0.826$, $p = 0.371$ for effect of PQ; $F_{(1, 27)} = 4.677$, $p = 0.040$ for effect of genotype, $n = 7\text{-}9$). **(F)** Quantification of MitoTimer red:green ratios demonstrated no significant differences in mitochondrial ROS levels in DA neuron projections to the SOG after PQ treatment ($F_{(1, 27)} = 0.143$, $p = 0.709$ for effect of interaction; $F_{(1, 27)} = 1.009$, $p = 0.324$ for effect of PQ, $F_{(1, 27)} = 0.418$, $p = 0.523$ for effect of PQ, $n = 7\text{-}9$). Data are represented as means ± SEM. Two-way ANOVA **(D, F)** with Tukey's multiple comparisons test **(E)**. $^*p < 0.05$.

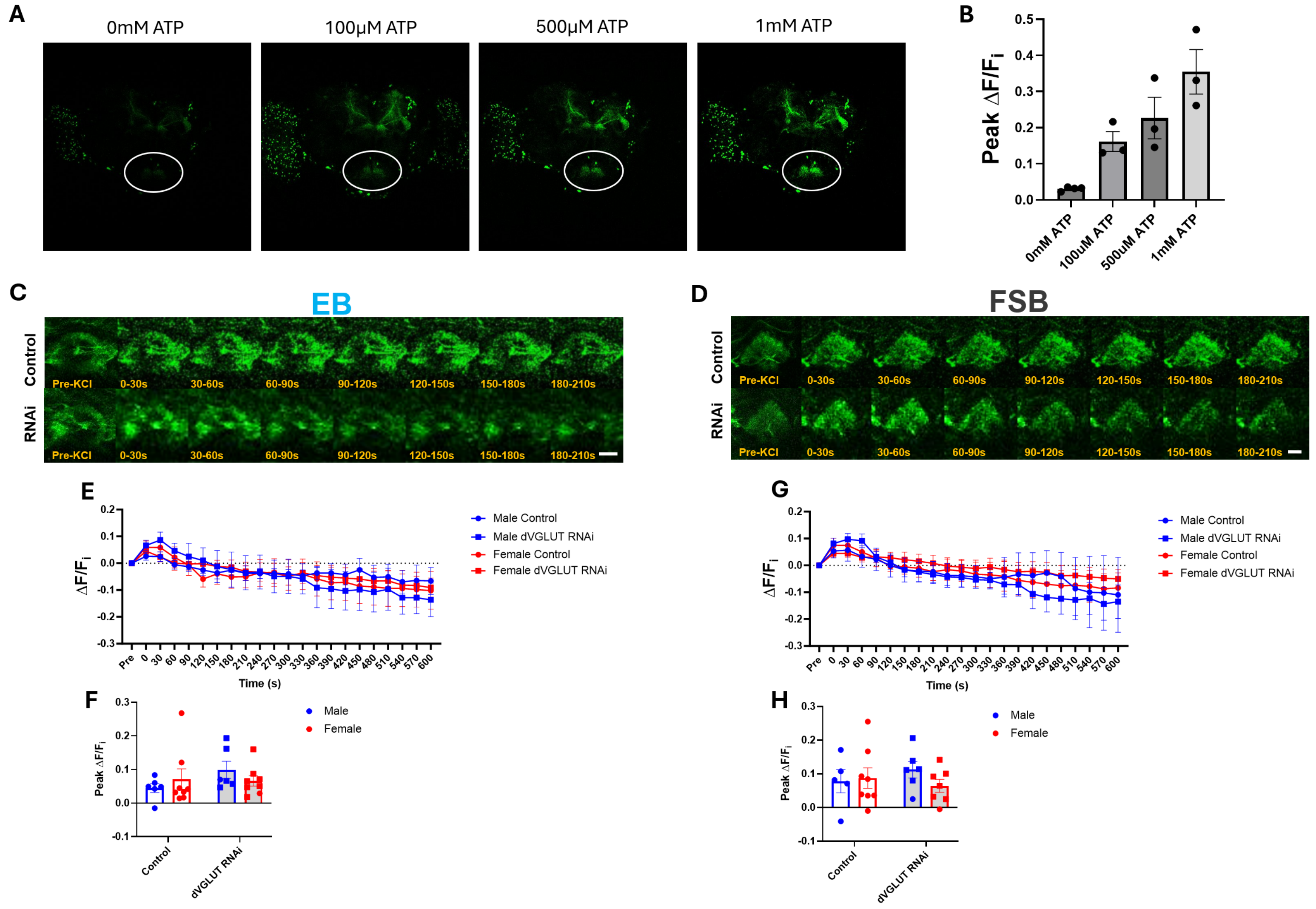

Figure S3. dVGLUT does not modulate activity-driven increases in intracellular ATP in dopaminergic projections to the EB or FSB. (A) Representative images of iATPSnFr-labeled dopaminergic projections to the SOG (white circles). **(B)** Quantification of iATPSnFr fluorescence in DA neuron projections to the SOG in response to perfusion of exogenous ATP revealed significant dose-dependent increases in iATPSnFr fluorescence ($F_{(3, 9)}$ = 11.94, p = 0.0017, n = 3-4). **(C-D)** Representative images of iATPSnFr-labeled dopaminergic projections to the EB **(C)** and FSB **(D)** in dVGLUT RNAi and control (LexA RNAi) flies. Scale bars = 25µm. **(E)** Quantification of iATPSnFr fluorescence in DA neuron projections to the EB before and after KCl-induced depolarization. **(F)** Quantification of peak iATPSnFr $\Delta F/F_i$ in projections to the EB demonstrated no changes in activity-driven intracellular ATP ($F_{(1, 24)}$ = 1.621, p = 0.216 for effect of interaction; $F_{(1, 24)}$ = 1.100, p = 0.305 for effect of genotype; $F_{(1, 24)}$ = 0.020, p = 0.8893 for effect of sex, n = 6-8). **(G)** Quantification of iATPSnFr fluorescence in dopaminergic projections to the FSB before and after KCl-induced depolarization. **(H)** Quantification of peak iATPSnFr $\Delta F/F_i$ in DA neuron projections to the FSB demonstrated no changes in activity-driven intracellular ATP ($F_{(1, 22)}$ = 1.054, p = 0.316 for effect of interaction; $F_{(1, 22)}$ = 0.041, p = 0.841 for effect of genotype; $F_{(1, 22)}$ =

0.480, p = 0.496 for effect of sex, n = 5-8). Data are represented as means ± SEM. One-way ANOVA **(B)**; Two-way ANOVA **(F, H)**.
